# Exploring the Influence of Planting Densities and Mulching Types on Photosynthetic Activity, Antioxidant Enzymes, and Chlorophyll Content and Their Relationship to Yield of Maize

**DOI:** 10.3390/plants13233423

**Published:** 2024-12-06

**Authors:** Li Zhao, Chang Zhang, Min Liang, Pei Chen, Sumera Anwar, Mingyuan Fan, Guangming Xie, Chuangyun Wang

**Affiliations:** 1 College of Agronomy, Shanxi Agricultural University, Taiyuan 030031, China; 19804643113@163.com (C.Z.); 15885155138@163.com (M.L.); cp13609475640@163.com (P.C.); f15187046458@163.com (M.F.); xiaoxiegua2401@163.com (G.X.); 2 Department of Botany, Government College Women University Faisalabad, Faisalabad 38000, Pakistan; sumeraanwar@mail.hzau.edu.cn

**Keywords:** *Zea mays* L., Loess Plateau, plastic mulch, straw mulch, physiology, grain yield

## Abstract

High-density maize cultivation can enhance yield, but water scarcity on the Loess Plateau may limit this potential. Mulching is a sustainable practice that conserves soil moisture, yet limited studies exist on the combined effects of planting densities and mulching types on maize performance in this region. Over two years, an experiment investigated the effects of mulching (no mulching (NM), plastic film mulching (PM), and straw mulching (SM)) at various densities (60,000 to 90,000 plants ha^−1^). The results showed that mulching significantly improved grain yield and other metrics compared to NM. PM and SM enhanced yields notably at 75,000 plants ha^−1^, while NM was more favorable at 67,500 plants ha^−1^. Physiological responses varied with density, showing a decreased photosynthesis rate alongside an increased transpiration rate. PM exhibited a 32 and 13% increase in catalase and superoxide dismutase activities, while malondialdehyde content was reduced by 7% compared to NM. The average of both years indicates that PM significantly increased the grain yield, net photosynthesis rate, and chlorophyll content by 5.8, 26.8, and 26.9%, while SM showed a 3, 12, and 12% increase, respectively, compared to NM. In conclusion, the combination of mulching and optimized planting density could enhance maize cultivation in the Loess Plateau.

## 1. Introduction

Mulching involves covering the soil with any organic or plastic material to conserve the moisture in the soil. It has been widely practiced in various tropical and temperate regions of China, Africa, the USA, and Australia to grow crops under limited soil water conditions [1]. Maize is a C4 thermophilic crop, and both low and high temperatures affect germination and subsequent growth and development processes [2,3]. Germination and early seedling growth require optimal soil temperatures [3]. Mulching plays a crucial role in this regard by moderating soil temperatures, providing insulation, and reducing temperature fluctuations [4,5]. By meeting the soil moisture and temperature requirements at each crop stage through mulching practices, maize photosynthesis and growth can effectively be coordinated and promoted, ultimately leading to higher yields and increased net economic returns [6].

In Loess Plateau regions, there is a growing demand for a high biomass production of maize and other crops, whether for fuel or animal feed [7]. Soil moisture is the most critical factor for the growth of rainfed crops in dryland areas due to limited water availability and seasonal and erratic rainfall [8,9]. The rainfed crops use the moisture stored during summer season rainfall. Typically, in this region, various materials, such as crop residues, husks, gravel, sand, and plastic films, have been used to cover the soil surface around crops to conserve soil moisture [10,11]. These mulching materials prevent moisture loss through evaporation and maintain soil temperature [4,11,12], especially during extreme weather conditions. Dong et al. [13] studied the effect of 13 years of straw mulching and plastic mulching during the maize growth period and plastic mulching during the fallow season and reported that all mulching practices effectively enhanced the soil moisture content by 12, 11, and 9%, respectively. Soil erosion caused by wind and water is the main ecological problem in agricultural dryland areas of the Loess Plateau. Studies have also shown that mulching helps to reduce soil erosion by reducing run-off and the direct impact of precipitation on soil [14,15]. Mulching also reduces nitrogen leaching to deeper soil layers [13] and suppresses weed growth, which competes with crops for water and nutrients, thereby improving crop yield and quality. This, in turn, promotes high biomass and healthier root growth and improves overall crop resilience to drought stress.

Most researchers suggest that plastic mulching is the most effective mulching practice for enhancing soil moisture in shallow soil layers. A meta-analysis study by Ma et al. [6] showed that plastic mulch increases the yield of spring maize by 79.4% and summer maize by 51.4% in the Loess Plateau areas. Similarly, Wang et al. [16] reported that 7 years of plastic mulching increased the grain yield of maize by >19% and also enhanced the WUE by >20%. Similar increases in yield and WUE by PM were also reported by Ren et al. [17] and Yang et al. [18].

Some of the previous researchers reported straw mulching as a feasible mulching approach that could replace plastic mulching due to the potential toxicity of plastic pollutants. Findings by Lan et al. [5] highlight that alternating straw mulching and no mulching beds demonstrate superior performance compared to plastic film mulching systems in improving silage yield and proposed it as a sustainable alternative for maize cultivation in semi-arid rainfed regions. Moreover, the decomposition of straw mulch materials enriches the soil with essential nutrients, contributing to soil fertility and health by enhancing organic matter content [19], microbial activity, and nutrient cycling, promoting long-term soil productivity and sustainability in rainfed agricultural systems.

Increasing plant density stands as the foremost strategy for enhancing maize yield. Maize crops exhibit some plasticity and resilience to the competitive pressures induced under high plant density by modifying the architecture traits, such as the plant height, internode length, leaf area, leaf angle, and ear position, which in turn affect the intercepted light, photosynthetic efficiency and yield [20,21]. However, for a rainfed crop, high plant densities under semi-arid and arid conditions can lead to extensive water depletion from soil [22]. Increased planting density can cause increased plant competition for light and water, which directly affects chlorophyll synthesis, resulting in less chlorophyll, a smaller leaf area, and a reduced plant yield [23].

Furthermore, high density causes shading, impeding light absorption [24], and stunting leaf development, thereby reducing photosynthesis rates, overall biomass production, and grain yield. Additionally, Gou et al. [25] observed a decline in maize leaf dimensions with increasing plant density. Zhao et al. [21] reported that increasing maize density above 67,500 plants ha^−1^ induces lodging by increasing the plant height and internode length and reducing the stalk diameter. This negative impact of dense planting arises from compromised photosynthetic activity and carbohydrate assimilation.

The effect of maize planting density on grain yield varies with the genotype [26,27], growing season [28], irrigation [29], and rainfall, especially for rainfed maize [22]. Dias et al. [26] identified optimal plant densities for hybrids DKB390 and BG7049YH at 78,500 plants ha^−1^ and 71,000 plants ha^−1^, respectively, based on trials ranging from 60,000 to 90,000 plants ha^−1^ in Brazil. Similarly, the optimal planting density varies with agroecological conditions. The planting density under rainfed conditions has been reported as being comparatively lower than that of irrigated maize [12,29].

The optimization of maize planting density under varying mulching practices presents a significant opportunity to address the dual challenges of water scarcity and yield stability in the Loess Plateau. Despite extensive studies on the individual benefits of mulching and planting density, their synergistic effects on physiological traits such as photosynthetic efficiency, antioxidative capacity, and water use efficiency remain poorly understood in this unique agroecological zone. By integrating advanced physiological assessments with local agronomic conditions, this study seeks to determine how specific mulching materials (plastic and straw) influence the viability of higher planting densities. We hypothesize that plastic and straw mulches could favor higher planting densities and yields by enhancing plant growth, water retention, and photosynthetic efficiency. This research not only offers a nuanced understanding of plant–environment interactions but also provides actionable insights for sustainable maize production systems tailored to the climatic and soil constraints of the Loess Plateau.

## 2. Results

### 2.1. Yield and Panicle Biological Traits

**Figure 1:**
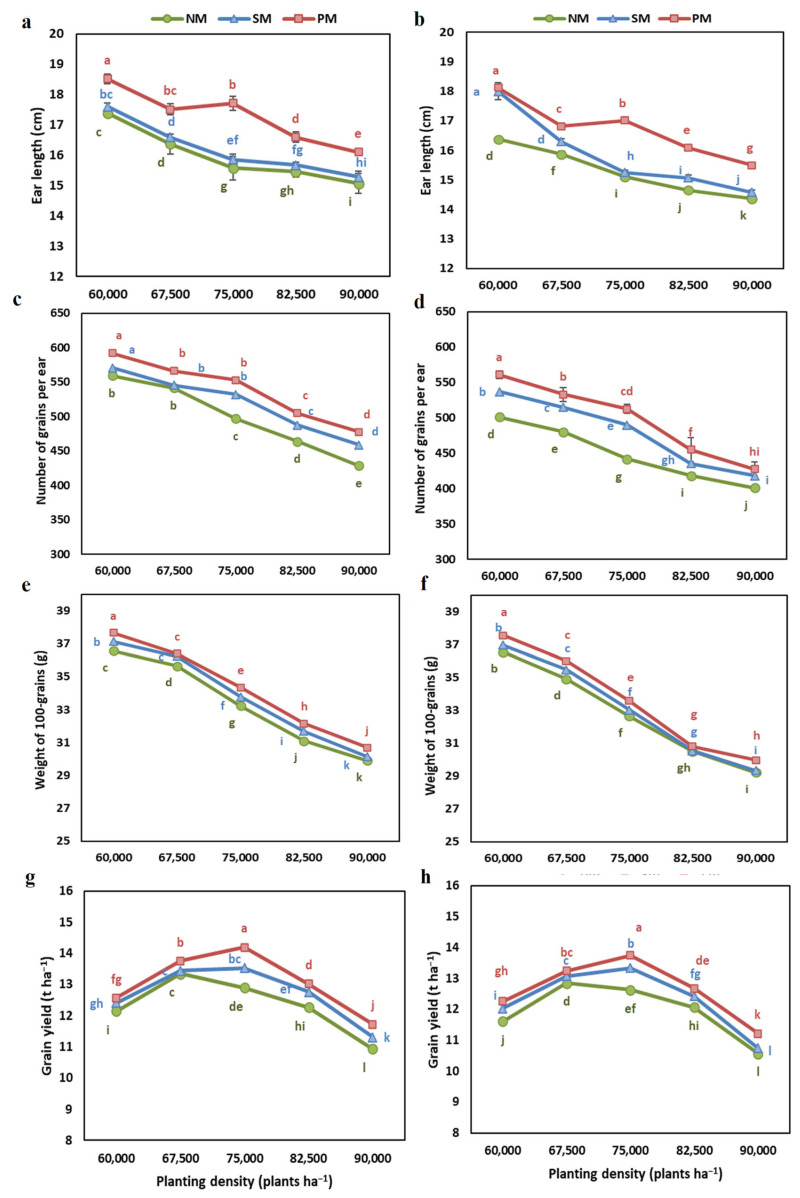
The interactive effect of planting density and mulching on (**a**,**b**) ear length, (**c**,**d**) number of grains per year, (**e**,**f**) 100-grain weight, and (**g**,**h**) grain yield of maize in 2022 (**a**,**c**,**e**,**g**) and 2023 (**b**,**d**,**f**,**h**). Different letters indicate significant differences among treatments by Tukey HSD test (*p* ≤ 0.05). NM: no mulching; PM: plastic mulching; SM: straw mulching.

The ear length, number of grains per ear, weight of 100 grains, and grain yield were recorded under different planting densities and mulching conditions in the years 2022 and 2023 (Table S1; Figure 1). The ear length was significantly affected by the mulching, density, years, and their interactions (Table S1). All yield traits were significantly higher in 2022 than in 2023. Among different mulching conditions, the ear length, number of grains per ear, the weight of 100 grains, and grain yield were maximum under PM, followed by SM, and were lowest under NM (Figure 1). Under PM, the ear length was 6–13% more in 2022 and 6–11% higher in 2023 than the NM under different planting densities. The number of grains per ear was 4.6–11.4% higher in 2022 and 6.6–16.1% higher in 2023. The weight of 100 grains was 2.1–3.4% higher in 2022 and 1.0–3.1% higher in 2023, and grain yield was 3.1–10% higher in 2022 and 3.1–9% higher in 2023 under PM than under NM.

Increasing planting density decreased ear length, number of grains per ear, and weight of 100 grains under all three mulching conditions. The effect of planting density on grain yield varied with mulching conditions. Under PM and SM, grain yield was highest at 75,000 plants ha^−1^, while under NM, grain yield was highest at 67,500 plants ha^−1^.

### 2.2. Net Photosynthetic Rate and Transpiration Rate

**Figure 2:**
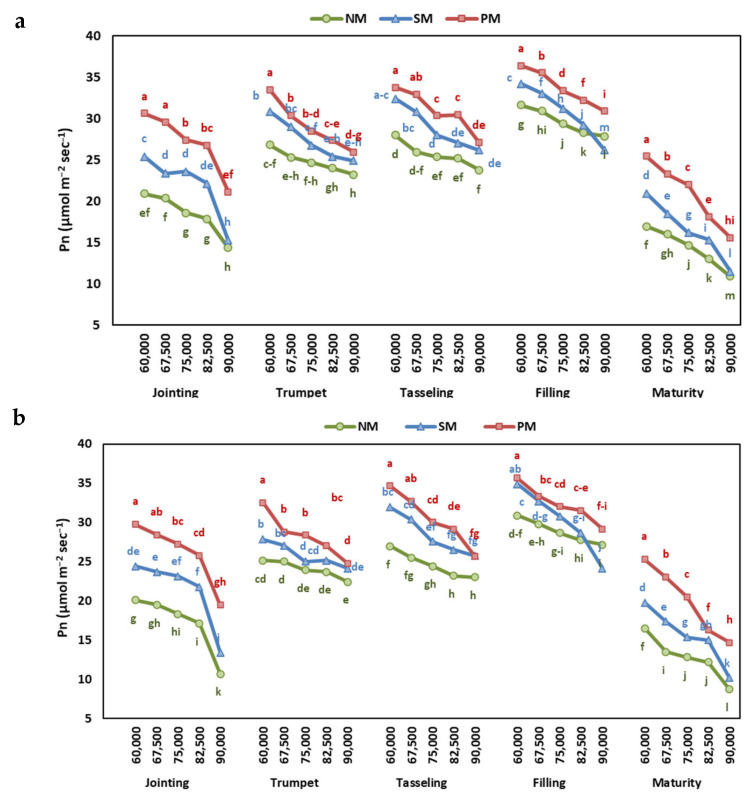
The effect of planting density and mulching on net photosynthesis rate at jointing, trumpet, tasseling, grain-filling, and maturity stages of maize in (**a**) 2022 and (**b**) 2023. Different letters indicate significant differences among treatments within a stage by Tukey HSD test (*p* ≤ 0.05). NM: no mulching; PM: plastic mulching; SM: straw mulching.

**Figure 3:**
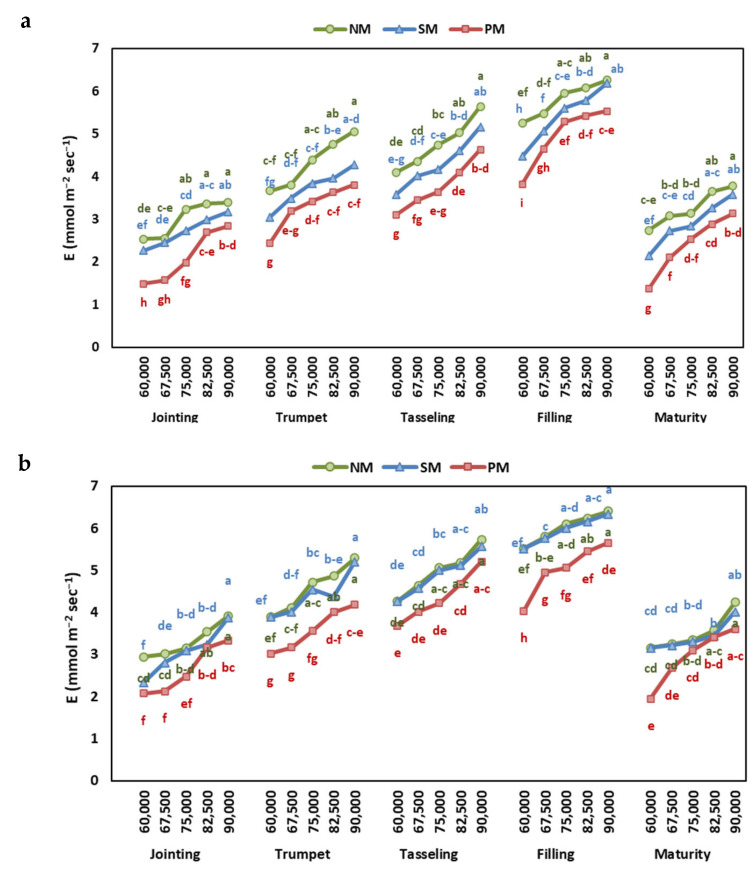
The effect of planting density and mulching on transpiration rate at jointing, trumpet, tasseling, grain-filling, and maturity stages of maize in (**a**) 2022 and (**b**) 2023. Different letters indicate significant differences among treatments within a stage by Tukey HSD test (*p* ≤ 0.05). NM: no mulching; PM: plastic mulching; SM: straw mulching.

The net photosynthesis (Pn) and transpiration rate (E) of maize leaf were determined at different growth stages and found to be significantly affected by the year, stages, mulching, and interaction (Table S2). The Pn and E increased with the growth stage, reached the maximum at the grain-filling stage, and then decreased at maturity (Figure 2 and Figure 3).

The increasing planting density decreased the Pn, which was 34% higher at 6000 plants ha^−1^ than at 9000 plants ha^−1^. In contrast, E increased with the increase in the density rate, and E increased by 39% at 9000 plants ha^−1^ compared to 6000 plants ha^−1^ (Table S2).

The Pn at all growth stages was highest under PM, followed by SM, and was lowest at NM (Figure 2). The E showed the opposite trend, with the highest E under NM and the maximum at PM (Figure 3). Overall, the Pn was highest at the grain-filling stage under PM at 6000 plants ha^−1^ and lowest under NM at 9000 plants ha^−1^ in both years.

### 2.3. Total Chlorophyll Content

The results of the effect of mulching and density rates on chlorophyll content in two years indicate significant variations among years, mulching types, density rates, and the interactions among stages, mulching, and density (Table S2). The chlorophyll content was highest under the PM treatment, followed by SM, and was lowest at NM. Among density rates, the highest chlorophyll content was observed at 67,500 plants ha^−1^ with a value of 5.15 mg g^−1^, closely followed by 75,000 plants ha^−1^ at 5.14 mg g^−1^. Conversely, the lowest chlorophyll content was recorded at 90,000 plants ha^−1^ with a value of 3.99 mg g^−1^. Across different stages of growth, the chlorophyll content was highest during the Jointing stage and gradually decreased towards maturity.

**Figure 4:**
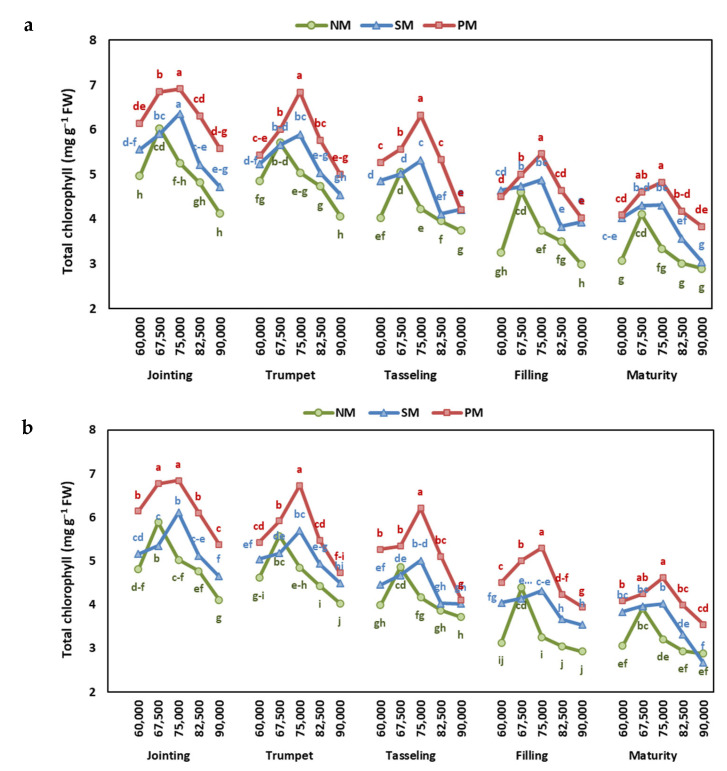
The effect of planting density and mulching on total chlorophyll at jointing, trumpet, tasseling, grain-filling, and maturity stages of maize in (**a**) 2022 and (**b**) 2023. Different letters indicate significant differences among treatments within a stage by Tukey HSD test (*p* ≤ 0.05). NM: no mulching; PM: plastic mulching; SM: straw mulching.

Overall, the highest chlorophyll content in both years was recorded at the trumpet stage under 75,000 plant ha^−1^ and PM, and the lowest was recorded at the maturity stage under 9000 plant ha^−1^ and NM (Figure 4).

### 2.4. Superoxide Dismutase and Catalase Activity

The results showed significant variations in catalase (CAT) activity and superoxide dismutase (SOD) activity across different stages, mulching types, and density rates (Table S2). The interactive effect of stage, mulching, and density rates was significant for CAT, and the interactive effect of mulching and density rates was significant for SOD.

**Figure 5:**
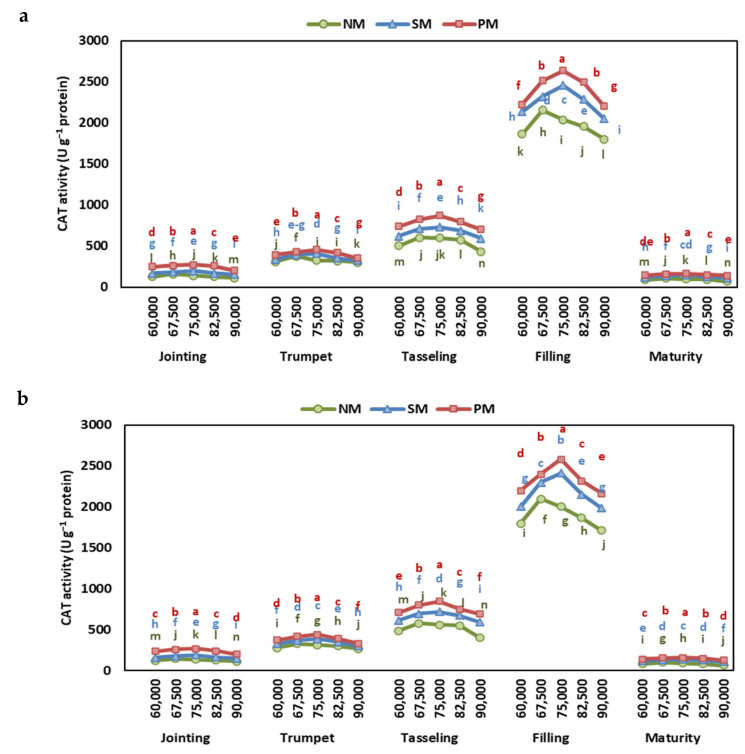
The effect of planting density and mulching on catalase activity at jointing, trumpet, tasseling, grain-filling, and maturity stages of maize in (**a**) 2022 and (**b**) 2023. Different letters indicate significant differences among treatments within a stage by Tukey HSD test (*p* ≤ 0.05). NM: no mulching; PM: plastic mulching; SM: straw mulching.

**Figure 6:**
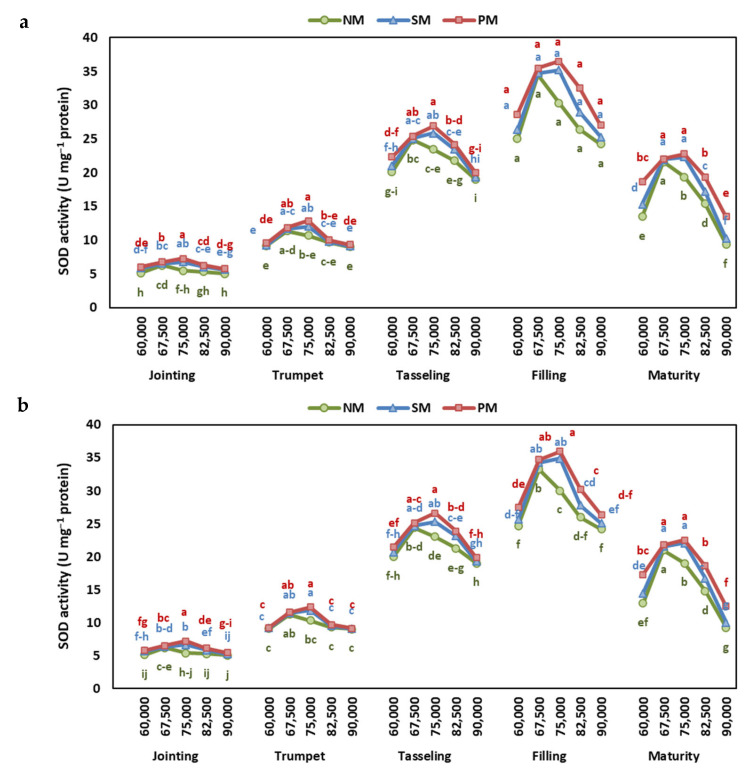
The effect of planting density and mulching on superoxide dismutase activity at jointing, trumpet, tasseling, grain-filling, and maturity stages of maize in (**a**) 2022 and (**b**) 2023. Different letters indicate significant differences among treatments within a stage by Tukey HSD test (*p* ≤ 0.05). NM: no mulching; PM: plastic mulching; SM: straw mulching.

In terms of CAT and SOD activity, the highest levels at all growth stages were observed under the PM, followed by SM and NM, in both years (Figure 5 and Figure 6). The average of both years showed that the CAT and SOD activities under PM were 38% and 13% higher than under NM (Table S2).

Increasing density rates first increased the CAT and SOD activity and then decreased. Under PM and SM, the highest CAT activity was recorded at 75,000 plants ha^−1^, while under NM, CAT, and POD, activities were highest at 67,500 plants ha^−1^. At the same time, the lowest CAT and POD activities were observed at 90,000 plants ha^−1^ under all mulching conditions.

### 2.5. Malondialdehyde Content

**Figure 7:**
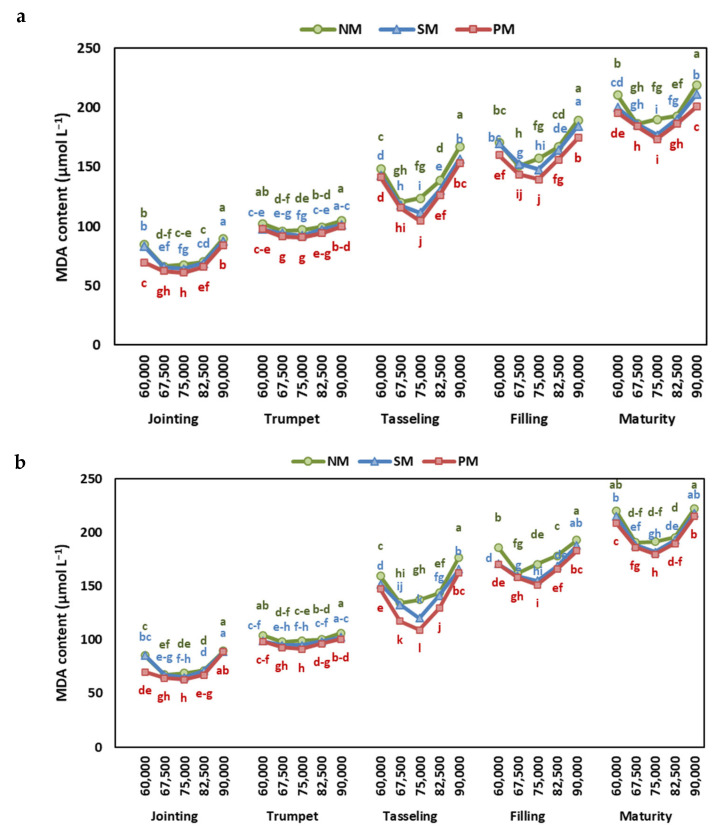
The effect of planting density and mulching on malondialdehyde (MDA) content at jointing, trumpet, tasseling, grain-filling, and maturity stages of maize in (**a**) 2022 and (**b**) 2023. Different letters indicate significant differences among treatments within a stage by Tukey HSD test (*p* ≤ 0.05). NM: no mulching; PM: plastic mulching; SM: straw mulching.

ANOVA results indicate significant differences in MDA content among years, stages, mulching types, density rates, and all their interactions. Higher MDA contents were observed in 2023 than in 2022. MDA content was highest under the NM treatment, followed by SM, and was lowest at PM. By increasing density rates, the MDA content showed a decreasing trend, reaching the lowest value at 67,500 plants ha^−1^ under NM or 75,000 plants ha^−1^ under SM and PM (Figure 7). Then, increasing plant density again increased the MDA content, reaching a maximum MDA of 9000 plants ha^−1^.

### 2.6. The Relationship Between Physiological Characteristics and Yield at the Grain-Filling Stage

**Figure 8:**
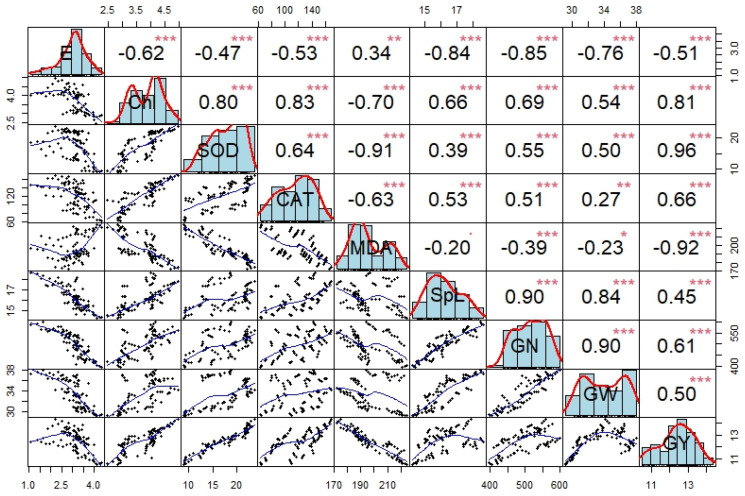
Correlation analysis among physiological and yield indices of maize at maturity. CAT: catalase activity; Chl: chlorophyll content; E: transpiration rate; GN: grain number per ear; GW: grain weight; GY: grain yield per hectare; MDA: malondialdehyde content; SOD: superoxide dismutase activity; SpL: spike length. *, **, and *** indicate significance at *p* ≤ 0.05, *p* ≤ 0.01, and *p* ≤ 0.001. Histograms (blue bars) on the diagonal display the data distribution for each variable, with red density curves overlaid. Scatter plots in the lower triangle illustrate relationships between variable pairs, while the upper triangle shows Pearson correlation coefficients. Asterisks (***) indicate levels of statistical significance (*p* < 0.05, * *p* < 0.01, ** *p* < 0.001).

Correlation indicates significant correlations of grain yield with grain weight, grain numbers per ear, spike length, catalase activity, superoxide dismutase activity, and chlorophyll content. It is also negatively correlated with transpiration rate and malondialdehyde content (Figure 8). The transpiration rate and malondialdehyde content were negatively correlated with all traits and positively related to each other.

## 3. Discussion

### 3.1. Effect of Mulching and Planting Density on Yield Traits

The study aims to elucidate the intricate relationship between physiological processes and yield components in maize under different mulching conditions. Plastic mulching emerges as a promising strategy for enhancing yield and physiological performance by creating favorable microclimatic conditions and minimizing environmental stress. Furthermore, careful consideration of planting density is essential to balance plant competition and resource allocation, thereby maximizing grain yield while maintaining physiological vigor.

The present results underscore the significant impact of mulching and planting density, and their interactions on the growth, development, and productivity of maize. The observed trends in yield-related traits, including ear length, number of grains per ear, weight of 100 grains, and grain yield, highlight the positive influence of mulching, particularly plastic mulching (PM), on overall productivity. Grain yield was 3.1–10% higher under PM than under non-mulched (NM) conditions. Similarly, the weight of 100 grains was 2.1–3.4% higher under PM than under NM.

This effect can be attributed to the ability of mulching to regulate soil temperature [5], moisture retention, and weed suppression, thereby creating optimal growth conditions for maize plants. Additionally, the superior performance of PM compared to straw mulching (SM) indicates that plastic mulches provide better insulation and moisture conservation, resulting in improved grain development and yield. These findings align with the previous research reporting higher maize grain yields under PM compared to SM, despite a higher moisture retention and silage yield by SM [5].

The grain yield exhibited an increasing and then decreasing trend with an increasing planting density of maize plants. The optimum planting density for maximum grain yield varied with mulching conditions. Under PM and SM, grain yield was highest at 75,000 plants ha^−1^, while under NM, it peaked at 67,500 plants ha^−1^. This contrasting effect of planting density on yield-related traits highlights the complex interplay between plant density and environmental factors. Optimal planting densities allow for maximum solar radiation interception and a balance between plant competition and resource availability [30]. The optimum increase in planting density favors the dry matter partitioning to the reproductive structures rather than to the vegetative organs at low-density rates [31]. The yield reduction at higher density rates occurs because of the depletion of water from soil at initial growth stages [22], inducing water deficiency and oxidative stress with a reduction in photosynthesis due to shading [27].

Soil moisture is the most limiting resource in the Loess Plateau under rainfed conditions [22]. Many studies indicate that plastic and straw mulching can effectively conserve moisture in the Loess Plateau region from summer rainfall [21]. Therefore, the conservation of soil moisture because of the reduction in evaporation allowed higher density rates under mulching (PM and SM), as compared to NM. Conversely, under NM, increasing planting densities at 67,500 plants ha^−1^ resulted in a reduction in grain yields, suggesting increased competition for water, nutrients, and light. Similarly, Liu et al. [12] mentioned that by applying plastic mulch, it was possible to raise the ideal maize plant population from 65,000 to 85,000 plants ha^−1^.

In contrast to grain yield, the increasing planting density showed a continuous decrease in ear length, number of grains per ear, and weight of 100 grains under all three mulching conditions. This indicates a reduction in yield-related traits at the optimum planting density at which the highest grain yield was achieved. This showed that the highest grain yield at a moderate planting density was not due to the increase in grain weight and grain yield per plant but because of the higher number of plants per unit area. Maize plants can adjust architectural and morphological traits in response to planting density [20,32]. A change in the plant height, leaf area, leaf thickness, leaf angle, stomatal abundance, and photosynthetic traits has been observed by various authors in maize by changing planting densities [32]. Higher planting density can induce shade avoidance mechanisms and lodging, reducing yield [20,21].

Grain number is affected by the light available per plant during a critical reproductive stage. Meanwhile, grain weight generally depends on the duration and rate of grain assimilation [33]. At higher density rates, plants face a reduction in light interception, which causes a reduction in photosynthesis [33]. Our results also indicate that the reduction in grain weight and grain number per ear is due to the reduction in the net photosynthesis rate, which causes less matter assimilation to reproductive parts.

These results indicate that yield initially increases with increasing planting density until the density reaches a level at which the available resources become too limited. The increase in yield per unit area becomes less than the reduction in grain weight and grain number per plant.

### 3.2. Effect of Mulching and Planting Density on Photosynthetic Capacity

Physiological parameters, such as net photosynthesis (Pn), transpiration rate (E), and chlorophyll content, exhibited differential responses to mulching and planting density, reflecting the complex regulatory mechanisms governing plant growth and development. The chlorophyll content and net photosynthesis (Pn) were higher under mulching than without mulch. The PM and SM enhanced the Pn by 26.7 and 12.1% and chlorophyll content by 26.9 and 11.6%, respectively, when compared to the NM. These results reinforce the role of mulches in enhancing photosynthetic efficiency and water uptake [34]. The higher chlorophyll content under PM and SM then contributes to higher biomass accumulation and, ultimately, grain yield [16,34]. Furthermore, mulching enhances the activity of antioxidant enzymes, which helps lower ROS and MDA levels, protects plants from oxidative damage, preserves photosynthesis, and delays senescence during drought conditions [34,35].

The highest chlorophyll content observed at 67,500 and 75,000 plants ha^−1^ underscores the significant impact of planting densities on chlorophyll content. These densities provide optimal conditions for resource allocation, leading to a higher chlorophyll content [36]. The highest chlorophyll content at the jointing stage reflects the vigorous vegetative growth phase, where photosynthetic activity peaks to support biomass accumulation. The gradual decrease in chlorophyll towards maturity aligns with the plant’s transition from vegetative to reproductive stages. During maturity, chlorophyll degradation occurs as resources are redirected to seed and grain filling [37].

A continuous decrease in Pn was observed as the planting density of maize increased from 6000 plants to 9000 plants ha^−1^. The observed decrease in Pn with an increasing planting density may be attributed to a greater competition for light and resources [27], leading to a reduced leaf area, leaf thickness, and photosynthetic capacity per plant [38,39]. Khan et al. [39] observed a reduced leaf area and leaf thickness of cotton with a lower intercellular CO_2_ concentration and stomatal conductance at a higher planting density.

The reduction in Pn at higher density rates during an increase in E indicates that the reduction in Pn is not linked to the reduced stomatal conductance or intercellular CO_2_ concentration. The reduction in Pn might be linked to the reduction in the capacity of mesophyll cells due to the reduction in chlorophyll content and oxidative damage under water deficiency. At the same time, the increased competition for light and nutrients further exacerbates the reduction in CO_2_ assimilation [40].

Many studies verified that PM significantly reduced evapotranspiration [41,42,43] while the ratio of transpiration to evapotranspiration increased [44]. At the same time, some studies reported no discernible effect of PM on maize evapotranspiration [45]. Some contradictory studies even reported higher transpiration rates of maize by PM in the semi-arid rainfed region of the Loess Plateau in China [42,45].

Furthermore, the E increased with the density rates, indicating that the reduction in Pn does not occur because of stomatal closure [2]. Conversely, the increasing transpiration rate at a high planting density suggests a compensatory mechanism to maintain photosynthesis and water uptake by keeping stomata open. Furthermore, at higher densities, the plants adjust the leaf angles to expose the upper leaves so as to intercept light, thus increasing transpiration [45,46]. The increase in E could be attributed to decreased evaporation during low radiance because of shading, which means that maize transpiration has increased while soil evaporation has decreased [46].

Similarly, Chen et al. [42] reported that increasing plant density from 60,000 to 90,000 plants ha^−1^ resulted in a large increase in evapotranspiration. We concur with Ren et al. [47], who found that the ability of a higher plant density to lower maize evapotranspiration depends on the difference between decreased soil evaporation and increased transpiration.

### 3.3. Effect of Mulching and Planting Density on Oxidative Stress

Reactive oxygen species (ROS) are byproducts of metabolic processes that increase in quantity during stress conditions. While ROS play important roles as signaling molecules in various physiological functions, their levels must be carefully regulated since excess ROS can damage cellular structures. Antioxidants play a critical role in maintaining redox homeostasis within biological cells by keeping ROS concentrations within safe limits, thus preserving the integrity of key macromolecules, such as lipid membranes, DNA, and proteins. Notably, antioxidant enzymes, such as catalase (CAT) and superoxide dismutase (SOD), facilitate the breakdown of harmful compounds, such as hydrogen peroxide and superoxide ions [34].

In recent research, it was found that the activities of CAT and SOD were significantly higher in mulched plants compared to those without mulching. Specifically, plants under PM demonstrated increases in CAT and SOD activities of 32% and 13%, respectively, while those under SM showed increases of 18% and 7% relative to NM. These enhanced activities of CAT and SOD suggest improved antioxidant defense mechanisms, likely due to reduced oxidative stress and better overall plant health. The lack of mulching contributed to increased oxidative stress, primarily caused by soil moisture deficiency, whereas mulching practices helped improve soil moisture levels and mitigate oxidative stress [35,47]. Reduced competition under mulching minimizes stress from resource scarcity, allowing plants to allocate more energy to antioxidant enzyme activity, thereby enhancing their ability to mitigate oxidative damage [48].

Furthermore, plants subjected to mulching exhibited lower malondialdehyde (MDA) content in leaves when compared to those grown without mulching. Specifically, PM and SM reduced MDA levels by 7% and 4%, respectively, relative to NM, indicating a reduction in lipid peroxidation and membrane damage. The elevation in CAT and SOD activities, coupled with decreased MDA levels, signifies that mulching effectively diminishes ROS production and the resultant damage [34,47].

Additionally, the levels of SOD and CAT activities in maize leaves were observed to increase before declining with higher planting densities throughout various growth stages. The specific planting density at which peak antioxidant enzyme activity occurred differed depending on the type of mulching. In no mulching conditions, maximum SOD and CAT activities were recorded at 67,500 plants ha^−1^, while for PM and SM, peak activities were noted at 75,000 plants ha^−1^. Previous research has also underscored the influence of planting density on antioxidant activity levels in plants. For instance, Wu et al. [40] reported similar trends of increasing and then decreasing SOD and CAT activities with escalating planting density in *Perilla frutescens*. Notably, the lowest MDA concentrations were observed at these optimal density levels, indicating that the increased antioxidant enzyme activities effectively mitigated ROS-induced damage [34,40].

## 4. Materials and Methods

### 4.1. Test Materials

The seeds of maize variety Dafeng 1407 were procured from Shanxi Dafeng Seed Industry Co., Ltd., Jinzhong, China. This variety is resistant to drought, lodging, silk smut, large spot disease, and ear rot. It has a growth period of about 126 days, cylindrical ears, red cobs, and yellow grains.

### 4.2. Overview of the Test Site

**Figure 9:**
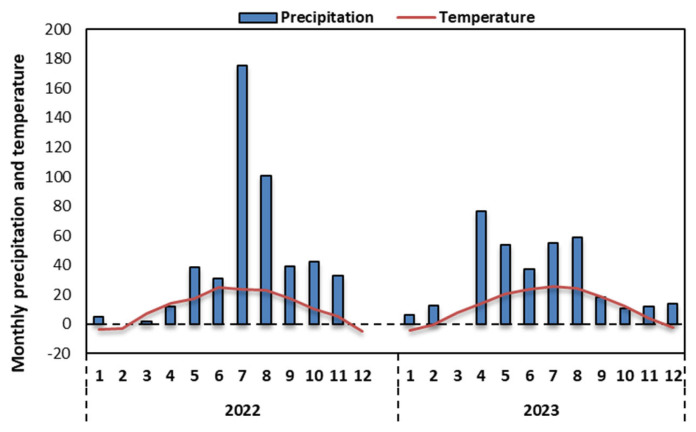
The average monthly temperature (°C) and monthly precipitation (mm) at the experimental site, Yuci station in Jinzhong, month by month.

This two-year experiment was conducted in 2022–2023 at the Dongyang Experimental Base (112°40′5″ E, 37°33′22″ N) of Shanxi Agricultural University. The test site has a warm-temperate semi-humid continental monsoon climate, with an average annual temperature of 9.7 °C, an average annual frost-free period of 158 days, and rainfall mainly concentrated in July to September. The total rainfall during 2022 and 2023 was 479 and 354 mm, respectively (Figure 9).

The soil of the experimental site was yellow clay. Soil properties were tested in 2022 and 2023. On 20 April 2022, the organic matter content of the soil in the 0–20 cm tillage layer was 9.96 g kg^−1^, the total nitrogen content was 101.24 mg kg^−1^, the available phosphorus content was 22.36 mg kg^−1^, the available potassium content was 152.16 mg kg^−1^, and the pH value was 8.08. On 20 April 2023, the organic matter content of the 0–20 cm tillage layer was 10.21 g kg^−1^; the total nitrogen content was 101.98 mg kg^−1^, the available phosphorus content was 23.41 mg kg^−1^, the available potassium content was 153.47 mg kg^−1^, and the pH value was 8.11.

### 4.3. Design and Management of Field Experiments

**Figure 10:**
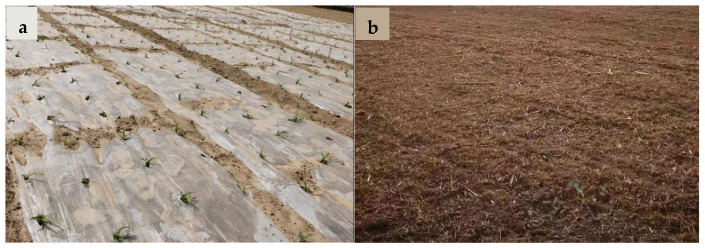
(**a**) Plastic mulching and (**b**) straw mulching in maize fields.

In this experiment, two factors (mulching and planting density) were arranged as a split-plot randomized block design. The three mulching methods were assigned to the main plots: no mulching (NM), plastic film mulching (PM), and straw mulching (SM). For PM, a 2 m wide polyethylene plastic film (Xifeng Plastic Co., Ltd., Baishan, China) with a thickness of 8 µm was used to cover the soil. For SM, the straw was left after harvesting the previous maize crop and then chopped and evenly distributed at the soil surface at a rate of 7500 kg ha^−1^ (Figure 10).

Seeds were sown on April 28 each year in equal row spacings 50 cm apart. Plant spacings were set as 33.4, 29.6, 26.7, 24.3, and 22.2 cm to attain a planting density of 60,000 plants ha^−1^ (P1), 67,500 plants ha^−1^ (P2), 75,000 plants ha^−1^ (P3), 82,500 plants ha^−1^ (P4), and 90,000 plants ha^−1^ (P5). Two to three seeds were sown in each hole, and the required density was maintained by thinning after germination. Each plot area was 40 m^2^, 10 m long, and 4 m wide, and each treatment was repeated 3 times. Every year, 1~2 days before sowing, compound fertilizer (N:P_2_O_5_:K_2_O in a ratio of 28:10:10) was applied at a rate of 750 kg ha^−1^ at the same time as rotary tillage with a fertilizer machine, and 300 kg ha^−1^ urea was top-dressed at the trumpet stage. Other field management measures are the same as in general fields.

### 4.4. Measurements and Growth Stages

All physiological traits were recorded at the jointing, trumpet, tasseling, filling, and maturity stages. The jointing stage (V6; BBCH17) was reached when the sixth leaf was fully unfolded, and the male tassel began to elongate. The corn trumpet stage (BBCH30) was reached when the corn plant had more than 10 visible leaves, more than 7 unfolded leaves, and upper leaves were rolled into a small trumpet shape 40 days after the corn seeds’ sowing. The tasseling stage (VT; BBCH63) was reached when the last branch of the male ear before silking was visible. The grain-filling milk stage (R3; BBCH 73) was reached when kernels had milky white fluid, and physiological maturity (R6; BBCH89) was reached when maximum dry matter accumulated, and the seeds were dry and hard with a black layer at the base of the grains.

### 4.5. Net Photosynthetic Rate, Transpiration Rate, and Total Chlorophyll Content

In the jointing, trumpet, tasseling, filling, and maturity stages, on a sunny day, the net photosynthetic rate (Pn) and transpiration rate (E) of representative functional maize leaves were determined from 8:00 am to 11:00 am by the LI-6400/XT Portable Photosynthesis System (LI-COR, Lincoln, NE, USA).

The same leaves used to determine photosynthesis were harvested and were used for the determination of chlorophyll content and antioxidant enzyme activities. A total of 0.5 g of leaves were crushed with 80% acetone, and the absorbance of the extract was determined at 663 and 645 nm by spectrophotometer (UV-1800 UV-Vis, Shimadzu Corporation, Kyoto, Japan). The total chlorophyll content was determined as suggested by MacKinney [49] and Arnon [50]:

(1) $$Total\;chlorophyll\;content=0.0202\;\times \;{ABS}_{645}\;+\;0.00802\;\times \;{ABS}_{663}$$

where ABS_645_ and ABS_663_ indicate the absorbance recorded at 645 nm and 663 nm wavelengths, respectively.

### 4.6. Antioxidants and Lipid Peroxidation

After harvesting, leaves were frozen directly into liquid nitrogen, and 0.5 g of frozen leaves were ground in 5 mL of extraction buffer consisting of 0.01 M phosphate buffer (pH 7.0) and 0.4% polyvinyl polypyrrolidone. The extract was then centrifuged at 10,000× *g* for 30 min.

For catalase (CAT) activity, 0.1 mL of enzyme extract was mixed with 1.9 mL phosphate buffer (pH 7.0) and 1.0 mL 0.075% H_2_O_2_ solution. CAT activity was determined spectrophotometrically by following the decline in ultraviolet absorbance at 240 nm using a spectrophotometer (UV-1800 UV-Vis, Shimadzu Corporation, Japan) as H_2_O_2_ was catabolized, according to the method of Beers and Sizer [51].

To determine superoxide dismutase (SOD) activity, plant extract (0.1 mL) was mixed with 63 µM nitroblue tetrazolium, 1.3 µm riboflavin, 13 mM methionine, and 0.05 M sodium carbonate (pH 10.2), and 3 mL final volume was attained with distilled water [52]. The samples were irradiated with a 15 W fluorescent lamp, and the photochemical reduction of nitrogen blue tetrazolium was detected at 560 nm using a spectrophotometer (UV-1800 UV-Vis, Shimadzu Corporation, Japan) [53].

The malondialdehyde content (MDA) was determined by the thiobarbituric acid colorimetric method [54]. The leaves (0.2 g) were homogenized in 5 mL 0.5% thiobarbituric acid and centrifuged at 10,000× *g* for 5 min. The 1 mL leaf extract reacted with 4 mL 20% trichloroacetic acid containing 0.5% thiobarbituric acid at 95 °C for 25 min. After cooling on an ice bath and centrifuging, the absorbance was read at 532 nm using a spectrophotometer (UV-1800 UV-Vis, Shimadzu Corporation, Japan), and the absorbance value at 600 nm was subtracted.

### 4.7. Yield Measurements

At the time of harvesting, the middle two lines of each plot were harvested to measure the yield. The length of corn ears (cm) was measured. After threshing and drying the cobs at 14% grain moisture, the weight of 100 grains (g) was recorded, and the number of grains per ear was counted. The seed test data were combined with the field yield measurement data to calculate the grain yield according to a grain moisture content of 14%.

### 4.8. Data Analysis

DPS version 7.05 (DPS Software Ltd., Enfield, UK) and Microsoft Excel 2007 (Microsoft Corporation, Redmond, WA, USA) were used for statistical analysis and for making experimental data figures. Correlation analysis was performed using RStudio Posit Software v. 2023.06.1 Build 524 (Posit, Boston, MA, USA).

## 5. Conclusions

The study investigated the impact of plastic and maize straw mulching and planting density on various yield traits, physiological parameters, and oxidative stress indicators in rainfed maize. Both types of mulching significantly enhanced the grain yield. Plastic mulching consistently outperformed straw mulching, resulting in higher yield traits. The highest grain yield was achieved at a planting density of 75,000 plants ha^−1^, at which plastic mulching showed a 10 and 9% higher grain yield and straw mulching showed a 4.8 and 5.7% higher grain yield than no mulching in 2022 and 2023, respectively. Mulching also mitigated oxidative stress, leading to a lower MDA content compared to no mulching. Increasing planting density from 6000 to 9000 plants ha^−1^ showed a non-linear quadratic trend in the grain yield ha^−1^, chlorophyll content, and antioxidant (SOD and CAT) activities of maize. These traits were first increased and reached the maximum at the optimal planting density (75,000 plants ha^−1^ under mulching and 67,500 plants ha^−1^ under no mulch) and then decreased. Meanwhile, the grain weight, grain numbers per ear, and photosynthesis rate linearly decreased with increasing density rates. Overall, plastic mulching emerges as a promising strategy for enhancing yield and physiological performance by creating favorable microclimatic conditions and minimizing environmental stress. Further research is warranted to explore the effect on different genotypes, sowing methods, and soil fertility to develop comprehensive management recommendations for sustainable maize production.

## Data Availability

The authors agree to share the data files and relevant material.

## Supplementary Materials

The following supporting information can be downloaded at: https://www.mdpi.com/article/10.3390/plants13233423/s1, Table S1. The ANOVA shows the main effects and F-values of the yield traits under different mulching and planting densities of maize; Table S2. The ANOVA shows the main effects and F-values of the physiological traits under different stages, mulching, and planting density of maize.
