# Supplementary material for: Exploring the Influence of Planting Densities and Mulching Types on Photosynthetic Activity, Antioxidant Enzymes, and Chlorophyll Content and Their Relationship to Yield of Maize
Source: Plants (Basel). 2024 Dec 6;13(23):3423. doi: 10.3390/plants13233423 (PMC11644714; doi:10.3390/plants13233423)
Supplement: Supplementary file 1 [file plants-13-03423-s001.zip › plants-3328856-supplementary.pdf]

Table S1. The ANOVA shows the main effects and F-values of the yield traits under different mulching and planting densities of maize.

**Treatments**		**Ear length (cm)**	**Number of grains per ear**	**Weight of 100-grains (g)**	**Grain yield (t ha^{-1})**
**Year**	2022	16.48 a	518.55 a	33.78 a	12.68 a
	2023	15.94 b	475.17 b	33.14 b	12.29 b
**Mulching**	NM	15.62 c	473.27 c	33.03 c	12.13 c
	SM	16.02 b	499.01 b	33.44 b	12.50 b
	PM	16.99 a	518.30 a	33.92 a	12.83 a
**Density**	60000	17.67 a	553.55 a	37.07 a	12.16 c
	67500	16.57 b	530.28 b	35.78 b	13.28 a
	75000	16.09 c	504.32 c	33.44 c	13.38 a
	82500	15.59 d	460.75 d	31.14 d	12.53 b
	90000	15.15 e	435.40 e	29.88 e	11.08 d
**ANOVA**	Y	477.41***	922.60***	100.73***	182.64***
	M	1075.97***	333.53***	64.78***	199.04***
	D	1217.02***	928.25***	1799.23***	839.03***
	Y x M	15.35***	4.63*	0.96	0.35
	Y x D	5.69***	1.63	5.52***	1.90
	M x D	34.87***	4.60***	0.72	8.07***
	Y x M x D	11.42***	4.72***	0.63	0.98

NM: no mulching; PM: plastic mulching; SM: straw mulching

Table S2. The ANOVA shows the main effects and F-values of the physiological traits under different stages, mulching, and planting density of maize.

Treatments		Pn (μmol m^{-2} sec^{-1})	E (mmol m^{-2} sec^{-1})	Chlorophyll (mg g^{-1} FW)	CAT (U g^{-1} protein)	SOD (U mg^{-1} protein)	MDA (μmol L^{-1})
**Year**	2022	25.31 a	3.79 b	4.76 a	710.6 a	17.36 a	131.74 b
	2023	24.39 b	4.18 a	4.56 b	683.8 b	16.96 b	137.16 a
**Stage**	Jointing	21.99 d	2.81 e	5.57 a	182.4 d	5.92 e	73.52 e
	Trumpet	26.57 c	3.99 c	5.23 b	356.4 c	10.24 d	97.87 d
	Tasseling	28.14 b	4.52 b	4.67 c	655.2 b	22.68 b	137.69 c
	Filling	30.90 a	5.53 a	4.11 d	2171.2 a	29.73 a	166.41 b
	Maturity	16.64 e	3.08 d	3.72 e	120.8 e	17.23 c	196.77 a
**Mulching**	NM	22.01 c	4.36 a	4.13 c	597.7 c	16.09 c	139.32 a
	SM	24.67 b	4.08 b	4.61 b	705.7 b	17.23 b	134.35 b
	PM	27.88 a	3.51 c	5.24 a	788.3 a	18.17 a	129.68 c
**Density**	60000	28.13 a	3.32 e	4.59 b	655.9 d	15.48 c	141.68 b
	67500	26.51 b	3.67 d	5.15 a	742.8 b	19.80 a	124.99 d
	75000	24.93 c	4.01 c	5.14 a	759.7 a	1 b9.70 a	122.63 e
	82500	23.76 d	4.29 b	4.44 c	702.6 c	16.81 b	132.06 c
	90000	20.92 e	4.63 a	3.99 d	625.1 e	14.01 d	150.90 a
**ANOVA**	Y	279.8***	411.9***	206.3***	6030.4***	5.42*	1437.9***
	S	8220.7***	2555.9***	2432.5***	4865674***	2548.1***	97370***
	M	3754.5***	657.4***	2119.5***	102559***	50.8***	1515.9***
	D	1964.4***	552.4***	1007.2***	21778.9***	185.1***	5453.1***
	S x M	104.8***	3.04**	15.9***	14670.5***	4.28***	53.3***
	S x D	48.4***	3.15***	7.14***	5023.2***	18.9***	274.6***
	M x D	40.2***	6.08***	98.7***	636.9***	3.2***	41.0***
	S x M x D	8.96***	1.93**	7.18***	258.3***	0.53	12.0***
	Y x S x M x D	1.86**	0.99	0.69	47.66***	0.03	3.46***

NM: no mulching; PM: plastic mulching; SM: straw mulching; Pn: net photosynthesis rate; E: transpiration rate; CAT: catalase activity; SOD: superoxide dismutase activity; MDA: malondialdehyde content.
